# Design, Synthesis, and Biological Studies of Flavone-Based Esters and Acids as Potential P450 2A6 Inhibitors

**DOI:** 10.1021/acs.chemrestox.3c00249

**Published:** 2023-11-14

**Authors:** Navneet Goyal, Camilla Do, Jayalakshmi Sridhar, Shahensha Shaik, Anthony Thompson, Timothy Perry, Loren Carter, Maryam Foroozesh

**Affiliations:** † Department of Chemistry, Xavier University of Louisiana, New Orleans, Louisiana 70125, United States; ‡ Cell and Molecular Biology and Bioinformatic Core, College of Pharmacy, Xavier University of Louisiana, New Orleans, Louisiana 70125, United States

## Abstract

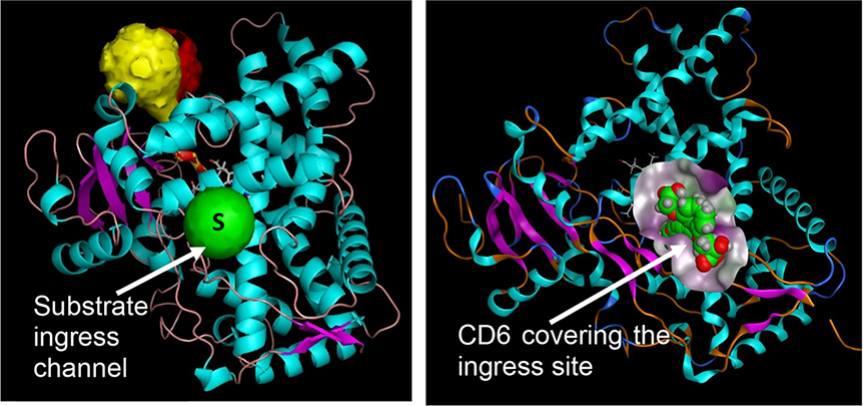

As a potential means for smoking cessation and consequently prevention of smoking-related diseases and mortality, in this study, our goal was to investigate the inhibition of nicotine metabolism by P450 2A6. Smoking is the main cause of many diseases and disabilities and harms nearly every organ of the body. As reported by the Centers for Disease Control and Prevention (CDC), more than 16 million Americans are living with diseases caused by smoking. On average, the life expectancy of a smoker is about 10 years less than a nonsmoker. Smoking cessation can substantially reduce the incidence of smoking-related diseases, including cancer. At least, 70 of the more than 7000 cigarette smoke components, including polycyclic aromatic hydrocarbons, N-nitrosamines, and aromatic amines, are known carcinogens. Nicotine is the compound responsible for the addictive and psychopharmacological effects of tobacco. Cytochrome P450 enzymes are responsible for the phase I metabolism of many tobacco components, including nicotine. Nicotine is mainly metabolized by cytochrome P450s 2A6 and 2A13 to cotinine. This metabolism decreases the amount of available nicotine in the bloodstream, leading to increased smoking behavior and thus exposure to tobacco toxicants and carcinogens. Here, we report the syntheses and P450 2A6 inhibitory activities of a number of new flavone-based esters and acids. Three of the flavone derivatives studied were found to be potent competitive inhibitors of the enzyme. Docking studies were used to determine the possible mechanisms of the activity of these inhibitors.

## 1 Introduction

Tobacco smoking is the most preventable cause of disease and mortality in the world today. According to the data published by the World Health Organization (WHO), tobacco use causes more than 7 million deaths per year, and if the pattern continues, it will cause more than 8 million deaths annually by 2030.^1,2^ Cigarette smoking is responsible for more than 480,000 deaths per year in the United States (US) alone, including the victims of secondhand smoke.^3^ In addition to the human health, suffering, and loss aspects, the total burden of smoking on the US economy is more than $300 billion per year, which includes over $200 billion in direct medical cost and $156 billion in lost productivity due to premature death and exposure to secondhand smoke.^4^

Smoking cessation has been shown to substantially reduce smoking-related mortality, even among long-term smokers.^5−7^ Less than 10% of smokers who attempt to quit smoking each year actually succeed.^6^ Because of the enormous health and economic costs of tobacco smoking, new treatment options need to be developed for dealing with the physical and psychological aspects of nicotine dependency to enable more individuals to successfully stop smoking.

Cytochrome P450 2A6 (CYP2A6) is an important member of the cytochrome P450 superfamily of heme-containing monooxygenases. These enzymes play an important role in the phase I metabolism of environmental toxicants, such as nicotine and other cigarette smoke components, into less toxic (detoxification) or more toxic (metabolic activation) metabolites.^8−14^ In addition to competitive inhibition, P450-dependent catalytic reactions are susceptible to mechanism-based inhibition by appropriate reactive “pseudosubstrates”.^15−20^ In humans, hepatic CYP2A6 accounts for approximately 85–95% of the metabolism of (S)-nicotine to cotinine.^21,22^ The P450-dependent metabolism of nicotine to cotinine by CYP2A6 proceeds through the (S)-nicotine Δ1′,5′-iminium ion, which is then converted to cotinine by cytosolic aldehyde oxidase.^10−14^ This iminium ion intermediate is a reactive electrophile capable of binding to microsomal macromolecules.^10^ It has previously been shown that selective mechanism-based inhibitors of CYP2A6 can be useful experimental tools to study the role of this enzyme in the metabolic activation of procarcinogens, such as 4-(methylnitrosamino)-1-(3-pyridyl)-1-butanone (NNK), to their ultimate carcinogenic forms.^17−20^

Previously, many groups including ours, have reported the design and synthesis of novel P450 2A6 inhibitors.^23−25^ Flavone and flavanone have been shown to be metabolized by P450 2A6 and to inhibit its catalytic activity.^26,27^ Here, we report the design, synthesis, biological, and modeling studies of a novel series of flavone-based esters and acids.

## 2 Materials and Methods

### 2.1 Syntheses

**Scheme 1:**
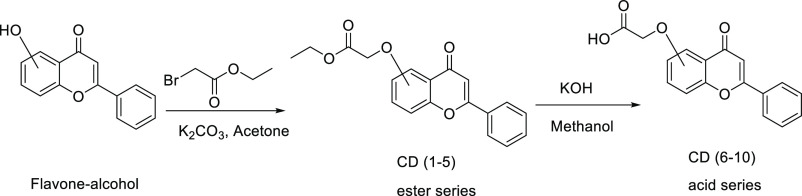
Synthesis Scheme for the Target Flavone-Based Ethyl Esters and Carboxylic Acids

**Figure 1:**
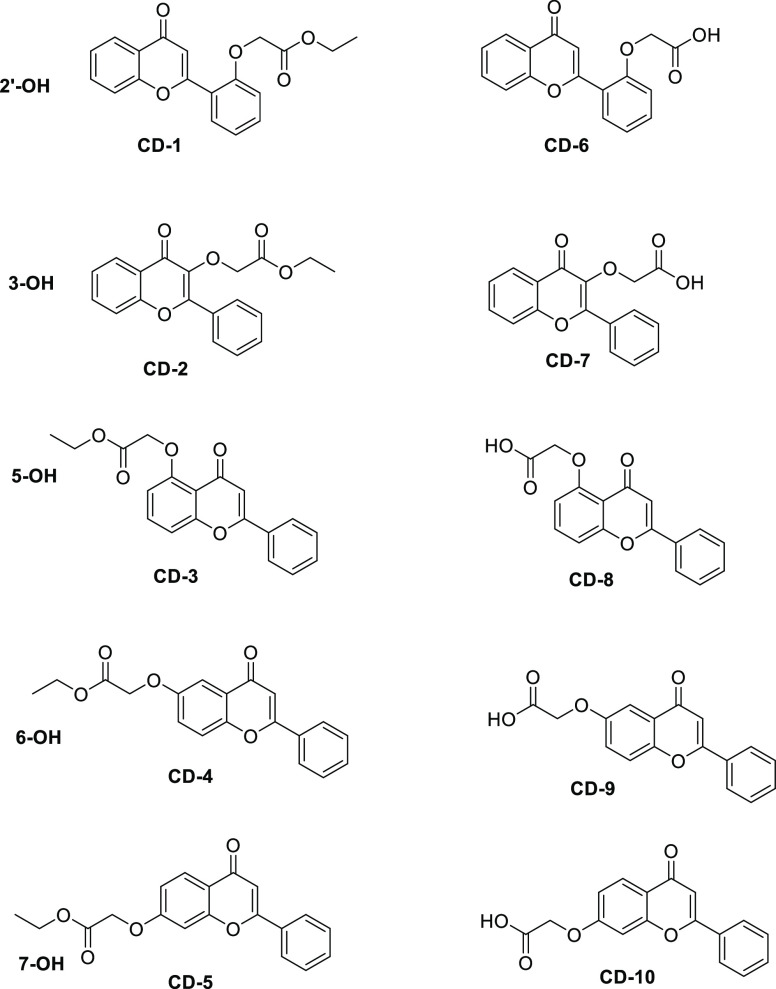
Structures of the flavone-based ethyl esters and carboxylic acids.

Flavone-based esters and corresponding acids were synthesized using commercially available flavone alcohols or phenols, as shown in Scheme 1. The commercially available flavone alcohol or phenol was dissolved in acetone in the presence of potassium carbonate (base), and reacted with bromoethyl acetate to obtain the ester. All esters were purified using combiflash chromatography, with hexane: ethyl acetate mixture as solvent, before hydrolyzation with potassium hydroxide (base) in methanol to get the corresponding carboxylic acid. All acid products were purified by recrystallization using ethyl acetate. No column chromatography purification was required for the acids. Structures of the esters and acids synthesized are shown in Figure 1.

#### 2.1.1 Compound 1 (CD-1): Ethyl 2-(2-(4-Oxo-4H-chromen-2-yl)phenoxy)acetate

(97% yield; yellow solid; mp 66–71 °C) ^1^H NMR (300 MHz, δ, ppm in CDCl_3_): 8.23 (d, *J* = 9.1 Hz, 1H), 7.93 (d, *J* = 8.1 Hz, 1H), 7.68 (d, *J* = 7.3 Hz, 1H), 7.56–7.38 (m, 3H), 7.24 (s, 1H), 7.16 (t, *J* = 7.6 Hz, 1H), 6.91 (d, *J* = 7.7 Hz, 1H), 4.76 (s, 2H), 4.28 (q, *J* = 7.2 Hz, 2H), and 1.31 (t, *J* = 7.2 Hz, 3H). ^13^C NMR (75 MHz, δ, ppm in CDCl_3_): 178.7, 168.1, 160.4, 156.4, 156.0, 133.5, 132.2, 129.6, 125.6, 124.9, 123.8, 121.7, 121.5, 118.0, 112.9, 112.5, 65.6, 61.6, and 14.0. *m*/*z* [M + H]^+^ for C_19_H_16_O_5_: calcd 325.1076, found 325.1068.

#### 2.1.2 Compound 2 (CD-2): Ethyl 2-((4-Oxo-2-phenyl-4H-chromen-3-yl)oxy)acetate

(99% yield; light yellow solid; mp 63–67 °C) ^1^H NMR (300 MHz, δ, ppm in CDCl_3_): 8.26 (d, *J* = 8.1 Hz, 1H), 8.22–8.18 (m, 2H), 7.73–7.68 (m, 1H), 7.58–7.51 (m, 4H), 7.44–7.39 (m, 1H), 4.91 (s, 2H), 4.18 (q, *J* = 7.2 Hz, 2H), and 1.23 (t, *J* = 7.2 Hz, 3H). ^13^C NMR (75 MHz, δ, ppm in CDCl_3_): 174.6, 169.1, 155.4, 155.2, 139.5, 133.6, 130.8, 128.9, 128.4, 125.8, 124.8, 124.0, 118.0, 68.3, 61.0, and 14.2. *m*/*z* [M + H]^+^ for C_19_H_16_O_5_: calcd 325.1076, found 325.1070.

#### 2.1.3 Compound 3 (CD-3): Ethyl 2-((4-Oxo-2-phenyl-4H-chromen-5-yl)oxy)acetate

(98% yield; white solid; mp 82–85 °C) ^1^H NMR (300 MHz, δ, ppm in CDCl_3_): 7.93–7.88 (m, 2H), 7.59–7.49 (m, 4H), 7.24–7.21 (m, 1H), 6.78 (d, *J* = 8.46 Hz, 1H), 6.75 (s, 1H), 4.84 (s, 2H), 4.30 (q, *J* = 7.2 Hz, 2H), and 1.31 (t, *J* = 7.2 Hz, 3H). ^13^C NMR (75 MHz, δ, ppm in CDCl_3_): 178.1, 168.6, 161.4, 158.4, 157.9, 133.6, 131.6, 131.5, 129.1, 126.2, 115.5, 111.9, 109.3, 109.2, 67.1, 61.6, and 14.3. *m*/*z* [M + H]^+^ for C_19_H_16_O_5_: calcd 325.1076, found 325.1069.

#### 2.1.4 Compound 4 (CD-4): Ethyl 2-((4-Oxo-2-phenyl-4H-chromen-6-yl)oxy)acetate

(83% yield; white solid; mp 111–115 °C) ^1^H NMR (300 MHz, δ, ppm in CDCl_3_): 7.96–7.90 (m, 2H), 7.58–7.52 (m, 5H), 7.42 (dd, *J* = 3.0, 9.0 Hz, 1H), 6.83 (s, 1H), 4.76 (s, 2H), 4.31 (q, *J* = 7.1 Hz, 2H), and 1.31 (t, *J* = 7.1 Hz, 3H). ^13^C NMR (75 MHz, δ, ppm in CDCl_3_): 178.0, 168.4, 163.4, 155.2, 151.6, 131.8, 131.6, 129.1, 126.3, 124.5, 124.3, 119.9, 106.9, 105.9, 65.6, 61.6, and 14.2. *m*/*z* [M + H]^+^ for C_19_H_16_O_5_: calcd 325.1076, found 325.1071.

#### 2.1.5 Compound 5 (CD-5): Ethyl 2-((4-Oxo-2-phenyl-4H-chromen-7-yl)oxy)acetate

(87% yield; white solid; mp 94–99 °C) ^1^H NMR (300 MHz, δ, ppm in CDCl_3_): 8.2 (d, *J* = 8.0 Hz, 1H), 7.94–7.90 (m, 2H), 7.57–7.51 (m, 3H), 7.10 (dd, *J* = 2.2, 8.9 Hz, 1H), 6.98 (d, *J* = 2.7 Hz, 1H), 6.81 (s, 1H), 4.84 (s, 2H), 4.33 (q, *J* = 7.2 Hz, 2H), and 1.34 (t, *J* = 7.2 Hz, 3H). ^13^C NMR (75 MHz, δ, ppm in CDCl_3_): 177.8, 168.0, 163.3, 162.2, 157.8, 131.8, 131.6, 129.1, 127.5, 126.3, 118.7, 114.3, 107.7, 101.7, 65.5, 61.8, and 14.2. *m*/*z* [M + H]^+^ for C_19_H_16_O_5_: calcd 325.1076, found 325.1072.

#### 2.1.6 Compound 6 (CD-6): 2-(2-(4-Oxo-4H-chromen-2-yl)phenoxy)acetic Acid

(72% yield; white solid; mp 181–185 °C) ^1^H NMR (300 MHz, δ, ppm in DMSO): 8.05 (d, *J* = 7.7 Hz, 1H), 7.97 (d, *J* = 8.1 Hz, 1H), 7.84–7.79 (m, 1H), 7.74–7.70 (m, 1H), 7.56–7.46 (m, 2H), 7.21–7.14 (m, 3H), and 4.90 (s, 2H). ^13^C NMR (75 MHz, δ, ppm in DMSO): 177.7, 170.3, 160.7, 156.5, 156.4, 134.7, 133.2, 129.7, 125.9, 125.2, 123.6, 121.8, 120.6, 119.0, 113.7, 112.5, and 65.4. *m*/*z* [M + H]^+^ for C_17_H_12_O_5_: calcd 297.0763, found 297.0759.

#### 2.1.7 Compound 7 (CD-7): 2-((4-Oxo-2-phenyl-4H-chromen-3-yl)oxy)acetic Acid

(66% yield; yellow solid; mp 145–148 °C) ^1^H NMR (300 MHz, δ, ppm in DMSO): 8.19–8.10 (m, 4H), 7.89–7.75 (m, 2H), 7.62–7.48 (m, 3H), and 4.81 (s, 2H). ^13^C NMR (75 MHz, δ, ppm in DMSO):174.4, 170.7, 155.3, 154.9, 139.6, 134.9, 131.6, 131.1, 129.3, 129.2, 125.8, 125.6, 123.9, 119.1, and 68.3. *m*/*z* [M + H]^+^ for C_17_H_12_O_5_: calcd 297.0763, found 297.0755.

#### 2.1.8 Compound 8 (CD-8): 2-((4-Oxo-2-phenyl-4H-chromen-5-yl)oxy)acetic Acid

(62% yield; light yellow; mp187–191 °C) ^1^H NMR (300 MHz, δ, ppm in DMSO): 8.08–8.03 (m, 2H), 7.68 (t, *J* = 8.2 Hz, 1H), 7.61–7.54 (m, 3H), 7.34 (d, *J* = 8.2 Hz, 1H), 6.99–6.81 (m, 2H), and 4.85 (s, 2H). ^13^C NMR (75 MHz, δ, ppm in DMSO): 177.3, 170.4, 162.9, 155.6, 151.1, 132.3, 131.6, 129.6, 126.8, 124.4, 124.1, 120.7, 106.6, 106.2, and 65.4. *m*/*z* [M + H]^+^ for C_17_H_12_O_5_: calcd 297.0763, found 297.0757.

#### 2.1.9 Compound 9 (CD-9): 2-((4-Oxo-2-phenyl-4H-chromen-6-yl)oxy)acetic Acid

(81% yield; white solid; mp 222–225 °C) ^1^H NMR (300 MHz, δ, ppm in DMSO): 8.11–8.07 (m, 2H), 7.76 (d, *J* = 9.5 Hz, 1H), 7.62–7.54 (m, 3H), 7.48–7.44 (m, 1H), 7.36 (d, *J* = 3.0 Hz, 1H), 7.0 (s, 1H), and 4.82 (s, 2H). ^13^C NMR (75 MHz, δ, ppm in DMSO): 178.2, 169.4, 165.4, 156.2, 152.6, 131.8, 131.2, 129.1, 125.3, 123.3, 119.9, 106.9, 106.2, and 64.5. *m*/*z* [M + H]^+^ for C_17_H_12_O_5_: calcd 297.0763, found 297.0760.

#### 2.1.10 Compound 10 (CD-10): 2-((4-Oxo-2-phenyl-4H-chromen-7-yl)oxy)acetic Acid

(43% yield; white solid; mp 216–220 °C) ^1^H NMR (300 MHz, δ, ppm in DMSO): 8.12–8.07 (m, 2H), 7.96 (d, *J* = 8.2 Hz, 1H), 7.62–7.55 (m, 3H), 7.32 (d, *J* = 2.5 Hz, 1H), 7.12–7.08 (m, 1H), 6.96 (s, 1H), and 4.88 (s, 2H). ^13^C NMR (75 MHz, δ, ppm in DMSO): 176.9, 170.0, 162.8, 162.7, 157.8, 132.2, 131.6, 129.6, 126.7, 118.0, 115.3, 107.3, 102.4, and 65.4. *m*/*z* [M + H]^+^ for C_17_H_12_O_5_: calcd 297.0763, found 297.0758.

### 2.2 P450 2A6 Inhibition Assays

Cytochrome P450 2A6 (CYP2A6) activity was determined using the Vivid CYP450 Screening Kit (Life Technologies, catalog no. PV6140) according to the manufacturer’s instructions. Briefly, a master premix containing baculosomes and regeneration system was prepared using 0.5× Vivid reaction buffer II. The test compounds were dissolved in dimethyl sulfoxide (DMSO) at a concentration of 100 mM. From the stock solutions, each compound was serially diluted in 0.5× Vivid reaction buffer II to make working stocks of 100, 50, 25 μM and so on up to 10 dilutions. It is essential to dilute the DMSO at least 1000-fold when making the working dilutions to prevent its interference with enzyme activity. An initial high-throughput screening was performed at a 100 μM concentration for each compound. Based on the results, the compounds that showed inhibition activity were selected for further studies. For the dose–response curves, in a 96-well plate, 40 μL of the diluted solutions of each test compound was added to each well, followed by 50 μL of the master premix, before incubation for 10 min at room temperature in the dark. A 10x mixture of Vivid substrate (reconstituted with acetonitrile) and NADP^+^ was then prepared. After the 10 min incubation period, to each well was added 10 μL of this solution to start the reaction. After 2 h of incubation in the dark at room temperature, the plate was read at 415 nm on a plate reader (Synergy H1, Biotek). For a positive inhibition control, tranylcypromine (Sigma-Aldrich, cat. no. P8511) was used at a final concentration of 100 μM. For a negative (no inhibitor) control, a 1:1000 dilution of pure DMSO in 0.5× Vivid reaction buffer was used. Each concentration in the dose–response curve was set up in triplicate, and each data point was the average of triplicate wells. The % inhibition was calculated using the following equation.
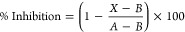
where *X* is the fluorescence observed in the presence of the test compound; *A* is the fluorescence observed in the absence of an inhibitor (no inhibitor control); and *B* is the fluorescence observed for the positive control. For graphing purposes, percent inhibition vs antilog [drug concentration] was plotted. A logistic sigmoidal model was used to fit the data and obtain IC_50_ values using Graphpad Prism software.

### 2.3 Docking Studies

Docking studies were performed using the Molecular Operating Environment (MOE) Program (Chemical Computing Group, Montreal, Canada).^23,28,29^ The coordinates of the template CYP2A6 (4EJJ.pdb)^30^ were taken from the Protein Data Bank (http://www.rcsb.org). Solvent molecules were removed, and hydrogen atoms were added to the template proteins using the Amber ff99 force field. Molecules CD-1 to CD-10 were built using the Builder module in MOE, and initial geometric optimizations of the ligands were carried out using the standard MMFF94 force field, with a 0.001 kcal mol^–1^ energy gradient convergence criterion and a distance-dependent dielectric constant employing Gasteiger and Marsili charges. Additional geometric optimizations were performed using the semiempirical method molecular orbital package (MOPAC). The catalytic site (occupied by nicotine close to the heme residue) was chosen as the initial docking site. A second site on the surface of the protein formed by the ingress/egress channel leading to the heme catalytic center was considered as an alternate binding site for the compounds in this study. Docking studies were performed for both binding sites, and the results were evaluated through visual inspection of the docked complexes.

## 3 Results and Discussion

### 3.1 P450 2A6 Inhibition Studies

**Figure 2:**
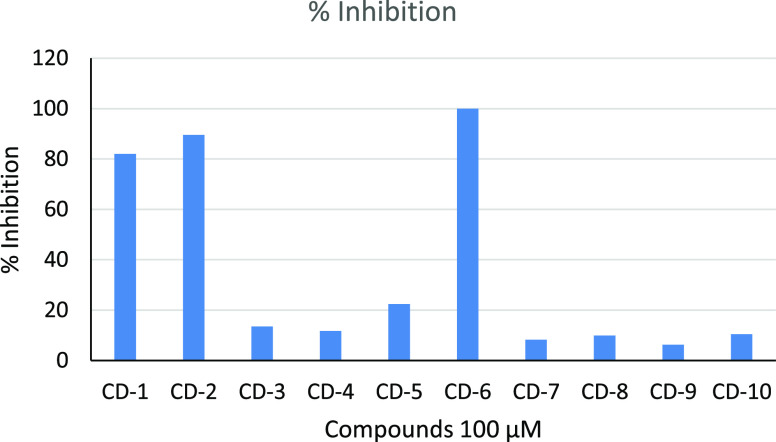
Percentage P450 2A6 inhibition activity of compounds CD-1 to CD-10.

Compounds CD-1 and CD-10 were tested at 100 μM for their inhibition of CYP2A6 (Figure 2). Compounds CD-1, CD-2, and CD-6 showed inhibition activities above 80%; with CD-6 inhibition at 100%. CD-5 showed 22% inhibition, while the other compounds did not show any significant inhibition activity.

**Figure 3:**
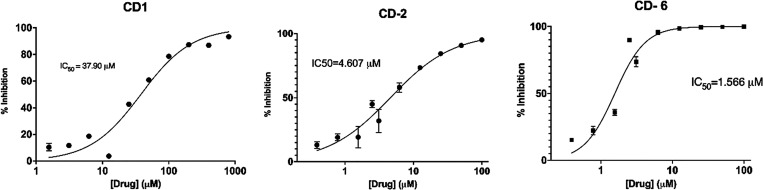
10-point dose–response curves for compounds CD-1, CD-2, and CD-6.

Dose–response curves for the most potent compounds, CD-1, CD-2, and CD-6, are shown in Figure 3. IC_50_ values for these compounds were determined to be 37.90 μM for CD-1, 4.607 μM for CD-2, and 1.566 μM for CD-6.

### 3.2 Docking Studies

**Figure 4:**
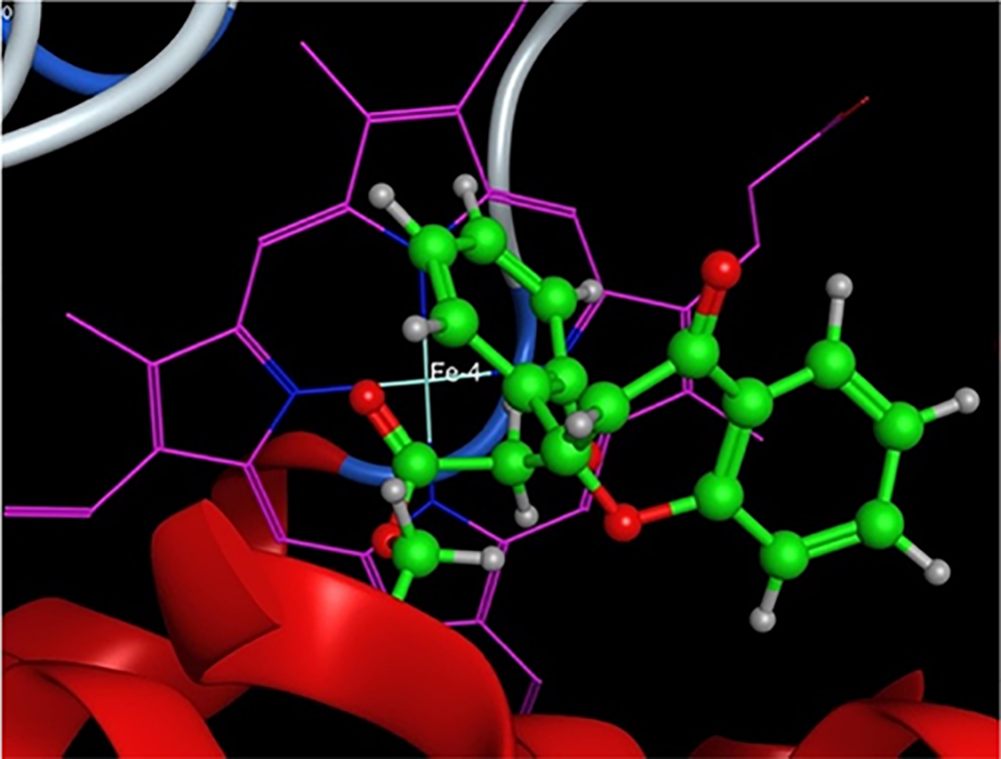
Compound CD-1 docked into the catalytic binding pocket of the CYP2A6 enzyme. Compound CD-1 is shown as a ball and stick model with carbons colored in green and the heme as a line model colored magenta. The distortion to the flavone ring can be clearly seen.

**Figure 5:**
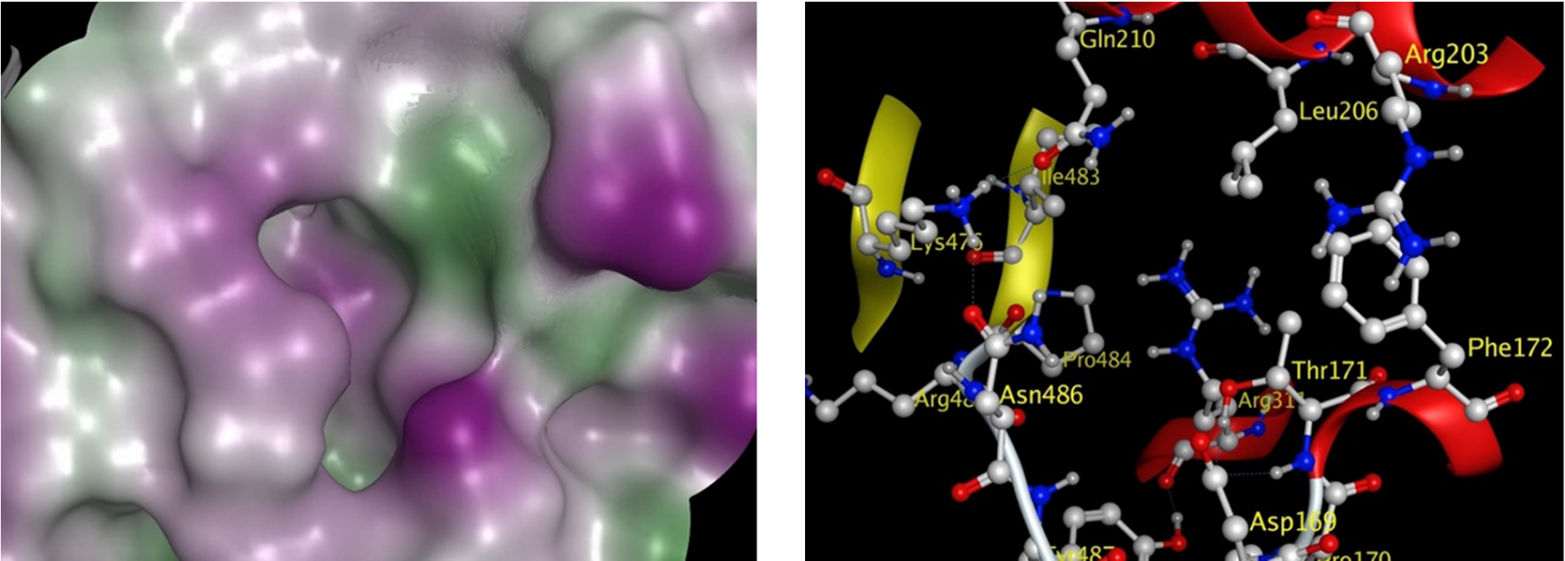
Surface view of the CYP2A6 enzyme ingress/egress channel to the catalytic site is depicted. (A) Molecular surface colored by lipophilicity (green for lipophilic and magenta for hydrophilic); and (B) The residues lining the ingress/egress site are shown as ball and stick models.

**Figure 6:**
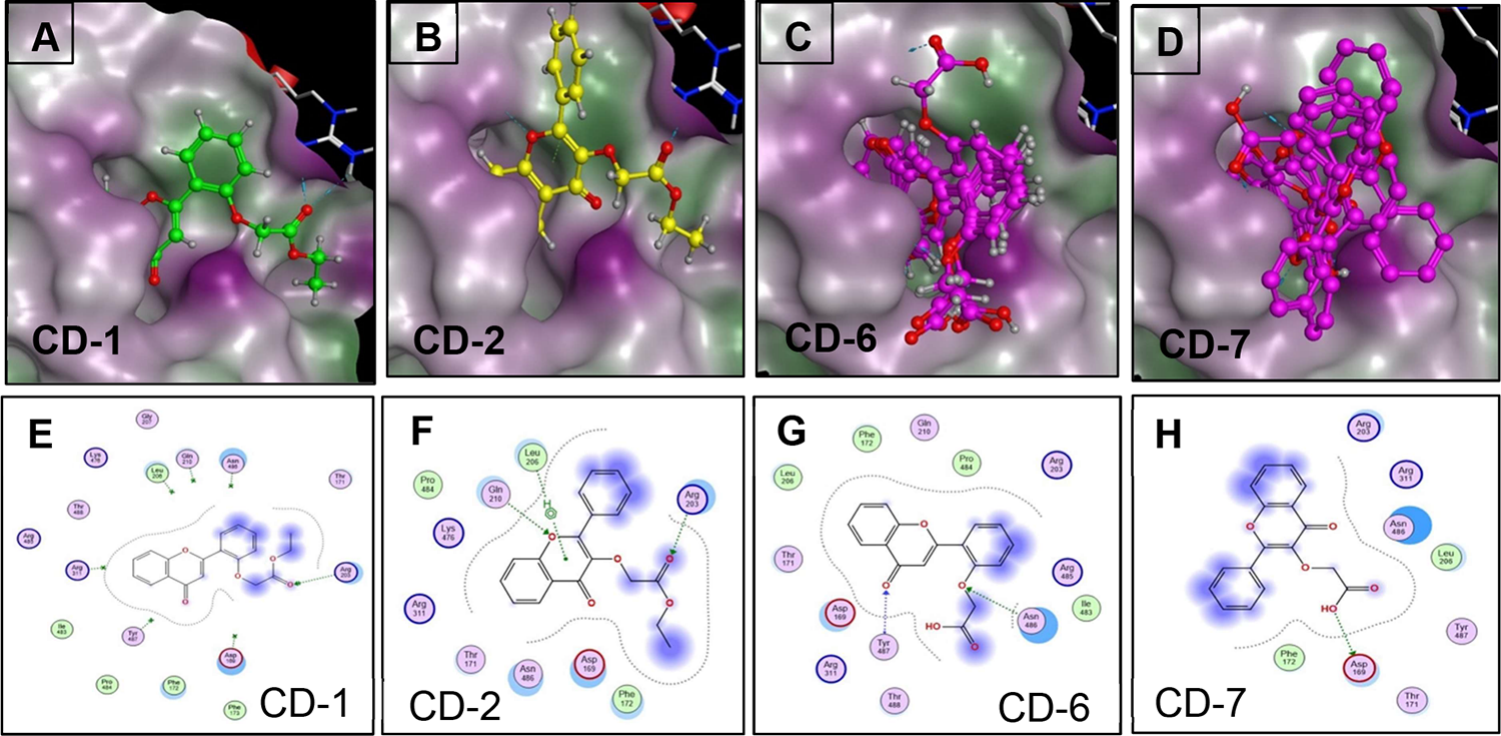
Binding modes of active compounds CD-1, CD-2, CD-6, and the inactive compound CD-7 from the docking studies on the enzyme surface ingress/egress channel site are depicted in (A–D). (A) Representative binding mode of compound CD-1; (B) Representative binding mode of compound CD-2; (C) All binding modes of compound CD-6; and (D) All binding modes of compound CD-7. The ligand interactions of compounds with the surrounding residues are shown in parts E–H for compounds CD-1, CD-2, CD-6, and CD-7.

The catalytic site of the CYP2A6 enzyme is small (281.7 Å^3^)^28^ when compared to the catalytic sites of CYP1A1 or CYP1A2 enzymes.^23^ The limitation presented by the size of the catalytic site can be evidenced in the structural size of the small molecules, such as nicotine, pilocarpine, and similar molecules, that are oxidized by the CYP2A6 enzyme. Docking studies of the compounds in the present study, CD-1 to CD-10, revealed that the molecules were folding up with the aromatic bicyclic ring and the side chain benzene ring no longer planar, and with visible distortions to their shapes (Figure 4). It was evident that the shape of the binding pocket and the orientation of many of the phenylalanine residues in the binding pocket caused distortion in the planar inhibitors when docked in the binding pockets. The Vivid kit used for analyzing the CYP2A6 inhibition by the compounds of this study works with a substrate that needs to be metabolized for detection. The assay relies on competitive binding of the ligands to the catalytic site. However, the docking studies clearly indicated that these compounds were too large to fit in the catalytic site of CYP2A6. A deeper speculation of this conundrum led to the assumption that if these molecules were too large to fit in the binding cavity and then to function as competitive inhibitors, they must have blocked the entry of the assay substrate into the catalytic site of the enzyme. In other words, these molecules bind to the surface of the enzyme in such a way that it effectively blocks the ingress-egress channel to the catalytic site of the CYP2A6 enzyme. The surface characteristics of the ingress/egress channel to the catalytic site is surrounded by several polar residues that include ASP169, THR171, GLN210, ARG311, LYS476, PRO484, ARG485, ASN486, and TYR487 (colored magenta in Figure 5). Only three nonpolar residues LEU206, ILE483, and PHE172 form a small lipophilic side of the pocket (colored green in Figure 5). This ingress/egress surface site was utilized for a second docking study of the compounds in this study (Figure 6). The binding modes of compounds CD-1, CD-2, and CD-6 were different from the binding modes of the rest of the compounds. For the active compounds CD-1, CD-2, and CD-6, the 4H-chromen-4-one part of the molecule resides in the inner part of the ingress/egress channel with hydrogen bonds formed by the oxygen atoms in the molecules and the site residues ARG203, GLN210, TYR487, and ASN486. Some compounds had H-pi interactions with LEU206. The compounds that were not active had the 4H-chromen-4-one part of the molecule exposed to the solvent with the side chain phenyl or the carboxyl group residing in the inner part of the ingress/egress channel, which led to lower interactions with the enzyme. While the docking studies indicate that blocking of the ingress/egress channel is a possibility, confirmation through mutational studies will be required.

## 4 Conclusions

The most potent inhibitor of cytochrome P450 2A6 in the series of flavon-based esters and carboxylic acids studied, CD-6 (IC_50_ of 1.57 μM), was the corresponding acid of the ethyl ester CD-1 (IC_50_ of 37.90 μM). The substituent for these two compounds is located on the 2′ position of the phenyl ring of the flavone moiety. Even though both compounds were active in the micromolar range, the acid was over 24 times more potent as a competitive inhibitor of this enzyme. The docking study results showed some important differences in the compounds’ binding modes with the enzyme surface ingress and exit channel site. Compound CD-6 made two hydrogen bonds with the site residues: the carbonyl of the flavone formed a hydrogen bond with the backbone nitrogen of Tyr487, and the phenolyic oxygen formed a hydrogen bond with the side chain amide nitrogen of Asn486. The carboxylic hydroxyl group of CD-6 was only 2.59 Å from the side chain carboxylic acid group of Asp169, which could potentially form a third hydrogen bond. Compound CD-1 formed only one hydrogen bond, with the ester carbonyl of CD-1 forming a hydrogen bond with the side chain nitrogens of Arg203. The additional hydrogen bonds made by CD-6 could be a contributing factor for the increased potency.

The other active inhibitor in this series, compound CD-2 (IC_50_ = 4.607 μM), has its ethyl ester substituent on position 3 of the flavone’s 4H-chromene-4-one double ring. Interestingly, its corresponding carboxylic acid, CD-7, is barely active. The docking study results showed critical differences in binding modes between these two compounds. The 4H-chromen-4-one part of CD-2 resided in the inner part of the ingress/egress channel, forming two hydrogen bonds. The ring oxygen formed a hydrogen bond with Gln210, and the ester carbonyl formed a hydrogen bond with Arg203. Additionally, there was H-pi interaction between the 4H-chromen-4-one and Leu206. All CD-7 binding modes showed the 4H-chromen-4-one part of the molecule out of the ingress/egress channel and exposed to the solvent, thereby not completely capping the channel. Only the side chain carboxylic acid group resided in the inner part of the channel, making a hydrogen bond to Asp169. The failure in complete capping of the channel by CD-7 could have contributed to its inactivity.

This study provided some interesting information regarding the mechanism of competitive inhibition of P450 2A6 by the compounds in this study. Since the enzyme is known to metabolize flavone and flavanone, the interactions seen for the flavone-based ethyl esters and acids must be due to the introduction of these substituents on the flavone ring system. Insertion of a larger group, other than a methyl group, prevents the molecules from fitting into the active site of cytochrome P450 2A6. Our previous study of introducing propargyl ether functional groups at the different positions of the flavone completely made them inactive against cytochrome P450 2A6 enzyme, while the P450 1A1 and 1A2 enzymes still metabolized them due to their larger active site.^31^ We hypothesize that the introduction of polar substituents on the flavone in this study provided the molecules with sufficient additional interactions with residues in the ingress/egress channel for them to be able to cap the channel, thereby inhibiting the metabolic activity of the enzyme. Our future work will involve confirming this hypothesis using two strategies. The first strategy will be to perform mutational studies on the enzyme amino acids that had hydrogen bonding interactions with CD-1, CD-2 and CD-6 to see if the inhibition activity of the compounds are reduced. The second strategy will focus on targeted modifications on these molecules toward increasing the interactions with the residues in the ingress/egress channel and studying their activity against the enzyme.
